# Perfusion scanning using ^99m^Tc-HMPAO detects early cerebrovascular changes in the diabetic rat

**DOI:** 10.1186/1756-6649-8-1

**Published:** 2008-03-13

**Authors:** Fatma J Al-Saeedi

**Affiliations:** 1 Nuclear Medicine Department, Faculty of Medicine, Kuwait University, Kuwait

## Abstract

**Background:**

^99m^Tc-HMPAO is a well-established isotope useful in the detection of regional cerebral blood flow. Diabetes gives rise to arterial atherosclerotic changes that can lead to significant end organ dysfunction, prominently affecting perfusion to the heart, kidneys, eyes and brain. In the current study, we investigated the role of ^99m^Tc-HMPAO cerebral perfusion scans in detecting early vascular changes in the diabetic brain.

**Methods:**

Cerebral perfusion studies were performed on both control and streptozotocin-(STZ) induced diabetic male Wistar rats. Rat brain imaging using a gamma camera was performed for each group 0.5, 2, 4, and 24 hours post ^99m^Tc-HMPAO injection. Data processing for each cerebral perfusion scan was performed by drawing a region of interest (ROI) circumferentially around the brain (B). Background (BKG) due to signal from the soft tissue of each rat was subtracted. Brain ^99m^Tc-HMPAO uptake minus background counts (net brain counts; NBC) were then compared between the two groups.

**Results:**

The NBC (mean ± SD) for the STZ group were statistically significantly higher (p = 0.0004) than those of the control group at each of the time points studied.

**Conclusion:**

^99m^Tc-HMPAO brain scan may be useful in the detection of early atherosclerotic changes in the diabetic rat brain.

## Background

Diabetes leads to early atherosclerotic changes through a number of mechanisms, including the formation of advanced glycosylation end products and its influence on serum lipid composition. The vascular complications of diabetes are significant, and can lead to a severe arteriopathy prominently affecting the heart, kidneys and eyes. The brain can also be affected, causing impairments in memory and learning.

Nuclear medicine studies have previously been utilized for the functional investigation of diabetic pathophysiology. For example, in the podiatric literature, nuclear medicine imaging has been shown to be helpful in the management of the diabetic foot [1]. The role of cerebral nuclear medicine imaging techniques in the detection of CNS manifestations of diabetes has been studied previously using ^18^F fluorodeoxyglucose (^18^F-FDG) positron emission tomography (PET), to assess brain glucose metabolism [2,3] in diabetic patients. Four studies have examined cerebral perfusion in diabetes using SPECT [4-7], three in type 1 diabetics and one in type 2 diabetics.

Technetium 99m-hexamethylpropylene amine oxime (^99m^Tc-HMPAO) cerebral perfusion scanning is a well-known nuclear medicine test used to detect variations in regional brain blood flow. ^99m^Tc-HMPAO is the most common radiotracer used for SPECT and planar brain imaging; it is a lipophilic radiotracer that crosses the blood brain barrier (BBB). ^99m^Tc-HMPAO is thought to accumulate in the brain through its intracellular conversion from a lipophilic to a hydrophilic form within the brain parenchyma [8]. Under most conditions, blood flow is coupled to increases in cerebral metabolism; hence ^99m^Tc-HMPAO images can be used to represent the functional status of the brain.

To our knowledge, no prior studies have investigated the use of ^99m^Tc-HMPAO uptake in the detection of vascular signal changes in early diabetic rats. Therefore, in the present study we investigated the potential role of ^99m^Tc-HMPAO cerebral perfusion scanning in the detection of early vascular changes in new-onset diabetes.

## Methods

### Materials

A HMPAO (Exametazime) kit was purchased from Amersham (UK). ^99m^Tc was eluted from a fresh ^99^Mo-^99m^Tc generator (Amersham, UK). All other reagents used in this study were supplied by Sigma-Aldrich (UK).

### Animals

Adult male Wistar rats (n = 12) of ~200 g weight were raised and handled in accordance with ethical standards, approved by the institutional ethics committee as recommended by the Helsinki Declaration.

### Preparation of ^99m^Tc-HMPAO

Fresh elutes of technetium (^99m^Tc) were used each time to prepare the ^99m^Tc-HMPAO following the manufacturer's instructions and recommendations. In brief, 1110–2960 MBq of ^99m^TcO_4 _in 5 ml of saline were added to a freeze-dried Exametazime kit to produce ^99m^Tc-HMPAO.

### Induction of experimental type 1 diabetes

Experimental type 1 diabetes was induced in rats by intraperitoneal (i.p.) injection of 55 mg/kg streptozotocin (STZ) dissolved in citrate buffer. Control rats were injected with buffer only.

### Blood glucose determination

Blood samples were collected from the tail vein. Basal glucose levels were determined prior to STZ injection, using an automated blood glucose analyzer (Glucometer Elite XL). Sample collections were then made 48 h after STZ injection and blood glucose concentrations were determined and compared between groups. Rats with blood glucose concentrations above 300 mg/dl were declared diabetic and were used in the experimental group. One week after the induction of experimental diabetes, imaging was performed.

### Experimental protocol

In order to control pain, an intravenous line was placed in the dorsal tail vein of each rat 10–15 minutes before the time of the radiopharmaceutical injection. Each rat was subsequently anaesthetized by intraperitoneal (i.p.) injection with 0.5 ml of 0.5 g intraval sodium 10 minutes before ^99m^Tc-HMPAO injection. The level of anesthesia achieved with this regimen lasted for ~4 hours. Another i.p. injection of 0.5 g intraval sodium was administered before imaging at the 24 hour point. This anesthetic agent is believed to have a negligible effect on both blood pressure and the biodistribution of the radiopharmaceutical. 129.5 MBq of ^99m^Tc-HMPAO was injected within 30 minutes of ^99m^Tc-HMPAO preparation and followed by a saline push administered via the fixed intravenous line. Each rat underwent a brain scan 30 minutes after ^99m^Tc-HMPAO injection.

### Gamma camera imaging

Each scan was performed using a single-head gamma (γ) camera (Philips camera; Odyssey LX) equipped with a high-resolution parallel hole collimator connected to a Dell computer. The matrix was 128 × 128 pixels and the photopeak was focused at 140 keV with a symmetric 10% window. Planar (posterior static view) images were obtained using a 5 minute acquisition at each time point. Each anaesthetized rat was fixed with plastic tape on a fixing board against the table of the γ camera during acquisition. The rat's head was localized in the center of the field of view and a zoom factor of 4 was applied during each acquisition time.

### Image processing

Data processing for brain scans was done by drawing a region of interest (ROI) circumferentially around the brain (B) and around the soft tissues of the neck of each rat as a background measurement (BKG). Brain ^99m^Tc-HMPAO uptake minus background (net brain counts; NBC) in control rats were determined first and expressed as the mean ± standard deviation (mean ± SD). NBC in diabetic-STZ rats were then determined. The ROIs were drawn with a fixed pixel size in order to maintain comparability between groups.

### Data presentation and statistical analysis

All data, unless otherwise stated, were expressed as the mean ± standard deviation (mean ± SD). A Kruskal-Wallis non-parametric analysis of variance (ANOVA) test was used to evaluate differences between the STZ diabetic group and the control group. In addition, differences between the two groups at each time point were assessed. Statistical analysis was performed using SPSS version 13.0 software (Chicago, USA).

## Results

Induction of diabetes by STZ resulted in a significant increase in blood glucose concentration (mean ± SD), from 180 ± 6 mg/dl (pre-STZ) to 350 ± 8 mg/dl (post-STZ) after one week of induction.

**Figure 1:**
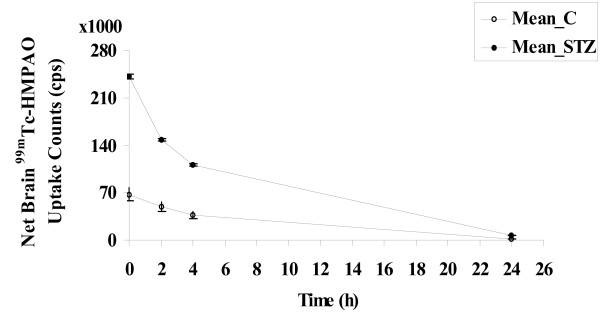
^99m^**Tc-HMPAO net brain counts (NBC; in cps) versus imaging time points: 0.5, 2, 4 and 24 h for control (mean ± SD): -○- Mean_C, open circles and diabetic-STZ (mean ± SD) group: -●- Mean_STZ, black circles.**

The net ^99m^Tc-HMPAO brain counts (NBC) in the control group (mean ± SD) at 0.5, 2, 4 and 24 h were 67,766 ± 10,405; 49,439 ± 6,960; 37,080 ± 5,459; and 2,017 ± 302 counts per second (cps) respectively. NBC for the STZ group at 0.5, 2, 4 and 24 h were 241,296 ± 3,548; 148,215 ± 2,316; 111,254 ± 1,682; and 6,817 ± 76 cps respectively. There was a highly significant difference between the control and STZ groups (p = 0.004) and there was no significant difference between each time point at both control and STZ groups (p = 0.416) using the non-parametric test equivalent to the analysis of variance (ANOVA), the Kruskal-Wallis test. Figure 1 shows ^99m^Tc-HMPAO net brain counts (NBC) for both control and STZ-treated rats.

## Discussion

Our results indicate that there are highly significant changes in the cerebral vasculature that occur early in the brains of diabetic rats, as demonstrated by an increase in ^99m^Tc-HMPAO NBC. We found evidence of significantly increased ^99m^Tc-HMPAO brain uptake one week after the induction of diabetes in the diabetic-STZ group compared to the control group at all time points from 0.5 h to 24 h. This may be due to early pathophysiological changes, such as vasodilatation in the early stages of diabetes associated with increased glucose metabolism. In agreement with our results, several prior studies have reported a significant increase in overall basal cerebral blood flow (CBF) rates in STZ-induced diabetic rats [9-11] as well as in diabetic patients using ^99m^Tc-HMPAO SPECT [4,5]. In contrast, one clinical study reported a 25–30% decrease in CBF in type 2 diabetic patients compared to control subjects [7]. These apparent inconsistencies may actually reflect time-dependent differences in the pathophysiologic course of diabetes; however, more definitive longitudinal studies are needed to evaluate this hypothesis.

Typically, blood flow parallels the rate of glucose metabolism in the cerebrum. The increased ^99m^Tc-HMPAO brain uptake observed in this study may be related to increased demand for, and/or accumulation of glucose uptake one week after the induction of diabetes.

^99m^Tc-HMPAO uptake was reported to accumulate significantly and to relate to an enhanced rate of glucose metabolism in malignant cancer cells such as in human breast tumor cell lines (MCF-7) *in vitro *[12]. This illustrates one manner in which CBF and glucose metabolism may be related. However, not all studies are consistent: in a group of patients with newly diagnosed type 1 diabetes, no changes in cerebral glucose metabolism were detected [13]. Furthermore, a group of patients with long-standing diabetes exhibited a 15–20% reduction in cerebral glucose metabolism, again possibly reflecting a time-dependent process [13]. Another potential confounding factor is the comparison of studies performed with different imaging modalities and radiopharmaceuticals, as the latter study was performed using [^18^F]-2-deoxy-2-fluoro-D-glucose (^18^F-FDG) PET [13].

## Conclusion

^99m^Tc-HMPAO brain scan may be useful in the early diagnosis of cerebral vasculopathic changes in early diabetic rats. Future studies should be performed to confirm our findings and to further delineate the relationship between diabetes and CBF characterized by ^99m^Tc-HMPAO.

## Competing interests

The author(s) declare that they have no competing interests.

## Authors' contributions

FA made substantial contributions to conception and design, data acquisition, analysis and interpretation, drafting and revisions of the manuscript.

## Pre-publication history

The pre-publication history for this paper can be accessed here:


